# Association of Vitamin D Status and COVID-19-Related Hospitalization and Mortality

**DOI:** 10.1007/s11606-021-07170-0

**Published:** 2022-01-01

**Authors:** Karen H. Seal, Daniel Bertenthal, Evan Carey, Carl Grunfeld, Daniel D. Bikle, Chuanyi M. Lu

**Affiliations:** 1 San Francisco Veterans Affairs Health Care System, Integrative Health Service, San Francisco, CA USA; 2 Departments of Medicine and Psychiatry, University of California San Francisco, San Francisco, CA USA; 3 Center of Innovation for Veteran-Centered and Value-Driven Care, VA Eastern Colorado Health Care System, Denver, CO USA; 4 Department of Biostatistics and Informatics, Colorado School of Public Health, University of Colorado Anschutz Medical Campus, Aurora, CO USA; 5 San Francisco Veterans Affairs Health Care System, Research Service and Division of Endocrinology and Metabolism, San Francisco, CA USA; 6 Department of Medicine, University of California San Francisco, San Francisco, CA USA; 7 San Francisco Veterans Affairs Health Care System, Division of Endocrinology and Metabolism and Dermatology, San Francisco, CA USA; 8 Department of Laboratory Medicine, University of California San Francisco and San Francisco Veterans Affairs Health Care System, San Francisco, CA USA

## Abstract

**Background:**

The relationship between vitamin D status and COVID-19-related clinical outcomes is controversial. Prior studies have been conducted in smaller, single-site, or homogeneous populations limiting adjustments for social determinants of health (race/ethnicity and poverty) common to both vitamin D deficiency and COVID-19 outcomes.

**Objective:**

To evaluate the dose-response relationship between continuous 25(OH)D and risk for COVID-19-related hospitalization and mortality after adjusting for covariates associated with both vitamin D deficiency and COVID-19 outcomes.

**Design:**

Retrospective cohort study.

**Patients:**

Veteran patients receiving care in US Department of Veteran Affairs (VA) health care facilities with a positive severe acute respiratory syndrome coronavirus 2 (SARS-CoV-2) test and a blood 25(OH)D test between February 20, 2020, and November 8, 2020, followed for up to 60 days.

**Main Measures:**

Exposure was blood 25(OH)D concentration ascertained closest to and within 15 to 90 days preceding an index positive SARS-CoV-2 test. Co-primary study outcomes were COVID-19-related inpatient hospitalization requiring airborne, droplet, contact, or other isolation and mortality ascertained within 60 days of an index positive SARS-CoV-2 test.

**Key Results:**

Of 4,599 veterans with a positive SARS-CoV-2 test, vitamin D deficiency (< 20 ng/mL) was identified in 665 (14.5%); 964 (21.0%) were hospitalized; and 340 (7.4%) died. After adjusting for all covariates, including race/ethnicity and poverty, there was a significant independent inverse dose-response relationship between increasing continuous 25(OH)D concentrations (from 15 to 60 ng/mL) and decreasing probability of COVID-19-related hospitalization (from 24.1 to 18.7%, *p*=0.009) and mortality (from 10.4 to 5.7%, *p*=0.001). In modeling 25(OH)D as a log-transformed continuous variable, the greatest risk for hospitalization and death was observed at lower 25(OH)D concentrations.

**Conclusions:**

Continuous blood 25(OH)D concentrations are independently associated with COVID-19-related hospitalization and mortality in an inverse dose-response relationship in this large racially and ethnically diverse cohort of VA patients. Randomized controlled trials are needed to evaluate the impact of vitamin D supplementation on COVID-19-related outcomes.

**Supplementary Information:**

The online version contains supplementary material available at 10.1007/s11606-021-07170-0.

The coronavirus disease 2019 (COVID-19) pandemic has resulted in unprecedented suffering, morbidity, and mortality worldwide.^1,2^ The COVID-19 vaccine has promised to decrease COVID-19 prevalence, yet persistent vaccine hesitancy and barriers to vaccine access in racial and ethnic minority and underserved populations coupled with emerging COVID-19 variants are leading to new surges of infection.^3–6^ Thus, interventions to mitigate COVID-19 disease severity remain highly relevant due to disparities in individuals’ ability to prevent or access effective therapies for COVID-19 and because of critical shortages of hospital beds.^7–9^

Vitamin D deficiency, typically defined as 25(OH)D < 20ng/mL,^10,11^ is widespread and considered a global public health problem.^12,13^ Vitamin D deficiency or low 25(OH)D is more prevalent in non-White individuals, those > 65 years and/or obese, and those residing in Northern latitudes with less sunlight. ^14–17^ Several studies have demonstrated an independent association between vitamin D deficiency and testing positive for severe acute respiratory syndrome coronavirus 2 (SARS-CoV-2).^18–20^

Other observational studies have reported poorer clinical outcomes from SARS-CoV-2 infection in patients with vitamin D deficiency, but these studies have been relatively small or single site.^21–23^ To date, randomized clinical trials of vitamin D supplementation on COVID-19-related outcomes have demonstrated mixed results.^24–26^ Moreover, vitamin D deficiency has been associated with a variety of chronic health conditions (e.g., diabetes, cardiovascular disease), yet randomized controlled trials have failed to demonstrate that vitamin D supplementation prevents or ameliorates these chronic conditions.^27–29^ This suggests that vitamin D may instead function as a marker for general health, nutritional status, and outdoor physical activity.^30^ A challenge in studying the relationship between vitamin D deficiency and COVID-19 outcomes is that risk factors for vitamin D deficiency are also associated with COVID-19 disease severity (e.g., obesity, medical comorbidities) as well as social determinants of health (e.g., non-Whiterace/ethnicity and poverty).^9,12,15,31^ Some prior COVID-19-related observational studies and trials have failed to fully adjust for potential confounding, particularly race/ethnicity, owing to the lack of diversity in the populations studied.^19,24,25^

Because of the controversy surrounding vitamin D supplementation, in July 2020, the US National Institutes of Health concluded “there are insufficient data to recommend either for or against the use of vitamin D for the prevention or treatment of COVID-19.”^32^ Using a Department of Veterans Affairs (VA) clinical database of veteran patients across the USA with positive SARS-CoV-2 tests linked to recent blood 25(OH)D test results, we investigated independent dose-response relationships between blood 25(OH)D concentrations and risk for COVID-19-related hospitalization and mortality. This study adds to the literature in including a large sample of geographically and racially and ethnically diverse patients, allowing adjustment for social determinants of health common to both vitamin D deficiency and COVID-19.

## METHODS

### Study Population

**Figure 1.**
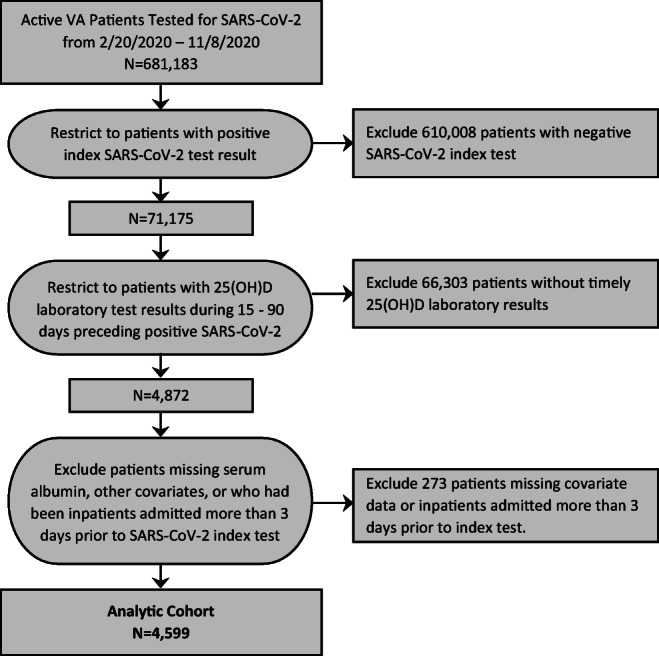
**Derivation of analytic cohort for patients with 25-hydroxy D laboratory results prior to positive SARS-CoV-2 test (2/20/2020–11/8/2020)**

This is a retrospective cohort analysis of veterans enrolled in VA health care systems across the USA tested for SARS-CoV-2 from February 20, 2020, to November 8, 2020, with up to 60 days of follow-up after the first or “index” SARS-CoV-2 test until study end (December 8, 2020) (Figure 1). Of 681,183 patients, 71,175 had positive SARS-CoV-2 tests, and of these, 4,872 had 25(OH)D tests within 15 to 90 days of their SARS-CoV-2 tests. Patients with 25(OH)D tests within 14 days of a positive SARS-CoV-2 test were excluded because vitamin D may act as a negative acute phase reactant, leading to reverse causality.^33^ Of these 4,872 patients, an additional 273 were excluded who were missing covariate data or were inpatients admitted more than 3 days prior to the SARS-CoV-2 test. This resulted in a final analytic cohort of 4,599 patients. This study was approved by the Committee on Human Research, University of California, San Francisco, and the San Francisco VA Health Care System Human Research Protection Program.

### Data Sources

The primary data source was the VA COVID-19 Shared Data Resource that included SARS-CoV-2 PCR test results, medical comorbidities associated with SARS-CoV-2 infection risk and disease severity (e.g., diabetes, chronic obstructive pulmonary disease (COPD), cardiovascular disease (CVD), smoking), health services utilization (including COVID-19-related hospitalization), and mortality data. The VA Corporate Data Warehouse (CDW) was linked to the COVID-19 Shared Data Resource to provide additional sociodemographic data and laboratory data, in this case, blood 25(OH)D and serum albumin test results. Data were prepared using SAS 9.4 (SAS Institute, Cary, NC),^34^ and analyzed using Stata 15.1 (StataCorp LP)^35^ software.

### Study Variables

#### Dependent Variables

Co-primary study outcomes were COVID-19-related inpatient hospitalization requiring airborne, droplet, contact, or other isolation and mortality ascertained within 60 days of an index positive SARS-CoV-2 test.

#### Independent Variables

Serum or plasma (heretofore “blood”) 25(OH)D test results in nanograms/milliliter (ng/mL) were ascertained closest to and within the 15 to 90 days preceding an index positive SARS-CoV-2 test. Blood 25(OH)D test results were obtained from more than 100 accredited VA clinical laboratories which use FDA-approved 25(OH)D assays (see Appendix Table 1). All assays are automated quantitative immunoassays measuring total blood 25(OH)D, including 25(OH)D2 and 25(OH)D3. These 25(OH)D assays have been standardized and compared to the reference ID-LC/MS/MS 25(OH)D assay traceable to the National Institute of Standards and Technology Standard Reference Material 2972.^36,37^ In binary analyses, vitamin D concentrations <20ng/mL represented vitamin D deficiency, consistent with clinical practice guidelines.^38^ Based on the distribution of 25(OH)D concentrations within the entire VA COVID database (*N*=237,934), 25(OH)D concentrations used in this study falling below the 5th percentile (i.e., 13.6ng/mL) and above the 95th percentile (i.e., 60.2ng/mL) were reset to 13.6 and 60.2 ng/mL respectively because small numbers of patients at either extreme could exert undue influence in a log-transformed model (see below).

Covariates in multivariable analyses included sociodemographics, such as age at index SARS-CoV-2 test date with relative risk calculated per 5-year increase; race/ethnicity; and poverty with relative risk calculated per 5% increase in the proportion of residents in patients’ zip code areas living below the federal poverty line.^39^ We included race/ethnicity and a proxy for poverty given these are two social determinants of health known to be associated with both SARS-CoV-2 infection and COVID-19 severity as well as vitamin D deficiency.^12,40^ Additional comorbidities associated with both COVID-19 severity and vitamin D deficiency were included, namely obesity (body mass index (BMI) as a continuous variable in kilograms/meters^2^ (kg/m^2^)), smoking, COPD, obstructive sleep apnea, CVD including hypertension, cancer, diabetes, chronic kidney disease (CKD), liver disease, human immunodeficiency virus (HIV), drug dependence, and alcohol dependence.^15,31^ The International Classification of Diseases, Tenth Edition (ICD-10) codes denoting each condition associated with VA outpatient and inpatient encounters that occurred within 2 years preceding the index SARS-CoV-2 test were included in these analyses.^41^

### Statistical Analyses

Descriptive univariate analyses were used to calculate the probabilities of individual associations between vitamin D deficiency (25(OH)D concentrations < 20 ng/mL) and sociodemographic factors and medical comorbidities, summarized as unadjusted pairwise relative risks (RR). Relative risks with *p*-values < 0.05 were considered significant. Both unadjusted and adjusted log-linked generalized linear models with Poisson distributions and cluster-robust error variances were used to estimate outcome probabilities and relative risks to describe the dose-response relationship between continuous 25(OH)D concentrations and COVID-19-related hospitalization and mortality.^42^ Since the relationship between 25(OH)D concentrations and the risk of outcomes may be non-linear, multiple functional forms of the model were investigated (*y*=*x*, *y*=*x*+ ln(*x*), *y*=*x*+*x*^2^, *y*=*x*+*x*^1/2^, *y*=*x*+*x*^1/3^, and *y*=*x*+*x*^1/4^). Given the potential for type 1 error inflation, resampling with replacement with 500 resamples was implemented for all functional forms of the model to reduce the impact of outliers and increase reproducibility of the results. The model with the consistently lowest average BIC across resamples was chosen as the final model functional form (*y*=ln(*x*)). Interactions between blood 25(OH)D and serum albumin (accounts for a small proportion of 25(OH)D binding)^43^ were tested and were not significant in any models. Sensitivity analyses that included serum albumin as a covariate did not significantly alter study findings (Appendix Tables 5 and 6).

Three generalized linear models were fit for each of the co-primary study outcomes (hospitalization and mortality): an unadjusted model with only log 25(OH)D concentration, a model adjusted for age and sex, and a model adjusted for all independent covariates. These continuous models were presented in terms of adjusted probabilities of the outcome—COVID-19-related hospitalization or mortality—conditional upon different blood 25(OH)D concentrations of interest. This involved using a post-estimation technique of predictive margins to calculate conditional probabilities and confidence intervals.^44^ In addition, relative risks for comparisons between outcome probabilities for any pair of blood 25(OH)D concentrations (e.g., 15 ng/mL vs. 20, 25, 30, 40, 50, or 60 ng/mL) could be made using margin contrasts, calculated as the ratio of the log of the margins followed by exponentiation.^44^

## RESULTS

Of 4,599 eligible patients with a positive index SARS-CoV-2 test and 25(OH)D measurement within 15 to 90 days, 588 (12.8%) were female, mean age was 62.6 years (SD +/−15.1 years); 1,578 (34.3%) identified as non-White, and 487 (10.6%) as Latinx (Table 1). The mean follow-up time was 54.9 days, with a range of 31 to 60 days. The median 25(OH)D concentration was 32.6 ng/mL (interquartile range=24.3–42.6 ng/mL) and 665 (14.5%) had 25(OH)D concentrations < 20 ng/mL, indicating vitamin D deficiency. In unadjusted analyses, Black or African American patients were at the very highest risk for low 25 (OH) D concentrations (RR=2.63; 95% confidence interval (CI)=2.28–3.04, *p*<0.001), as well as patients residing in areas with higher concentrations of poverty and those with alcohol and drug use dependence (Table 1).

**Table 1.** Factors Associated with Vitamin D Deficiency in 4,599 Patients with Positive SARS-CoV-2 and Recent 25(OH)D Result (2/20/2020–11/8/2020)

	All patients		Adequate vitamin D (≥20 ng/mL)		Vitamin D deficiency (<20 ng/mL)				
			*N*=3,934 (%)		*N*=665 (%)		Relative risk (95% CI)		*p-values*
**Sociodemographic factors**									
Age, mean (SD), y^a^	62.6	(15.1)	63.7	(14.8)	56.2	(15.3)	0.88	(0.86, 0.90)	<0.001
Sex									
Male	4,011	87.2%	3,453	87.8%	558	83.9%			
Female	588	12.8%	481	12.2%	107	16.1%	1.31	(1.08, 1.58)	0.005
Race									
White	3,021	65.7%	2,720	69.1%	301	45.3%			
Black or African American	1,171	25.5%	864	22.0%	307	46.2%	2.63	(2.28, 3.04)	<0.001
American Indian or Alaska Native	38	0.8%	32	0.8%	6	0.9%	1.58	(0.75, 3.33)	0.222
Asian	35	0.8%	29	0.7%	6	0.9%	1.72	(0.82, 3.59)	0.150
Native Hawaiian or Pacific Islander	40	0.9%	35	0.9%	5	0.8%	1.25	(0.55, 2.87)	0.589
Unknown	294	6.4%	254	6.5%	40	6.0%	1.37	(1.00, 1.86)	0.047
Ethnicity									
Not Hispanic or Latinx	4,112	89.4%	3,546	90.1%	566	85.1%			
Hispanic or Latinx	487	10.6%	388	9.9%	99	14.9%	1.48	(1.22, 1.79)	<0.001
Percent of residents < federal poverty line, mean (SD)^b^	16.2	(9.2)	16.0	(9.1)	17.4	(9.5)	1.07	(1.03, 1.11)	<0.001
**Medical comorbidities**									
Body mass index									
BMI Under 18	46	1.0%	42	1.1%	4	0.6%			
BMI 18–24	704	15.3%	628	16.0%	76	11.4%	1.24	(0.48, 3.24)	0.660
BMI 25–29	1,435	31.2%	1,264	32.1%	171	25.7%	1.37	(0.53, 3.53)	0.516
BMI 30–34	1,312	28.5%	1,110	28.2%	202	30.4%	1.77	(0.69, 4.56)	0.234
BMI 35+	1,102	24.0%	890	22.6%	212	31.9%	2.21	(0.86, 5.69)	0.099
Low serum albumin									
Normal serum albumin	4,049	88.0%	3,487	88.6%	562	84.5%			
Low serum albumin	550	12.0%	447	11.4%	103	15.5%	1.35	(1.12, 1.63)	0.002
Diabetes (any type)									
No	2,544	55.3%	2,163	55.0%	381	57.3%			
Yes	2,055	44.7%	1,771	45.0%	284	42.7%	0.92	(0.80, 1.06)	0.267
Cardiovascular dis. (incl. hypertension)									
No	2,712	59.0%	2,293	58.3%	419	63.0%			
Yes	1,887	41.0%	1,641	41.7%	246	37.0%	0.84	(0.73, 0.98)	0.023
Obstructive sleep apnea									
No	2,976	64.7%	2,567	65.3%	409	61.5%			
Yes	1,623	35.3%	1,367	34.7%	256	38.5%	1.15	(0.99, 1.33)	0.061
Chronic obstructive pulmonary disease									
No	3,612	78.5%	3,044	77.4%	568	85.4%			
Yes	987	21.5%	890	22.6%	97	14.6%	0.62	(0.51, 0.77)	<0.001
Cancer									
No	3,452	75.1%	2,915	74.1%	537	80.8%			
Yes	1,147	24.9%	1,019	25.9%	128	19.2%	0.72	(0.60, 0.86)	<0.001
Chronic kidney disease									
No	3,611	78.5%	3,077	78.2%	534	80.3%			
Yes	988	21.5%	857	21.8%	131	19.7%	0.90	(0.75, 1.07)	0.230
Liver disease									
No	4,214	91.6%	3,605	91.6%	609	91.6%			
Yes	385	8.4%	329	8.4%	56	8.4%	1.01	(0.78, 1.30)	0.960
Human immunodeficiency virus									
No	4,542	98.8%	3,891	98.9%	651	97.9%			
Yes	57	1.2%	43	1.1%	14	2.1%	1.71	(1.08, 2.72)	0.022
**Health risk behaviors**									
Smoking status									
Never smoker	1,904	41.4%	1,594	40.5%	310	46.6%			
Current or former smoker	2,695	58.6%	2,340	59.5%	355	53.4%	0.81	(0.70, 0.93)	0.003
Non-alcohol drug dependence									
No	4,393	95.5%	3,773	95.9%	620	93.2%			
Yes	206	4.5%	161	4.1%	45	6.8%	1.55	(1.18, 2.02)	0.001
Alcohol dependence									
No	4,153	90.3%	3,579	91.0%	574	86.3%			
Yes	446	9.7%	355	9.0%	91	13.7%	1.48	(1.21, 1.80)	<0.001

^a^Relative risk reflects 5 years increase in age.

^b^U.S. Census Bureau. American Community Survey, 2018 American Community Survey 5-Year Estimates. Table 1901. Accessed October 2, 2020. https://data.census.gov/cedsci/. Relative risk reflects 5% increase in proportion of residents in patient ZIP code living below federal poverty line.

Of the 4,599 patients with positive SARS-CoV-2 tests in this cohort, 964 (21%) were hospitalized for COVID-19 infection; of these, 146 (15.2%) had vitamin D deficiency <20 ng/mL. As shown in Table 2, in the fully adjusted model, there is a highly significant inverse relationship between increasing continuous 25(OH)D concentrations and decreasing risk for COVID-19-related hospitalization (*p*= 0.009). Other covariates independently associated with an increased risk for COVID-19-related hospitalization included increasing age (13% per 5-year increase in age), non-White race (50% increase), diabetes (24%), CVD (27%), cancer (21%), chronic kidney disease (44%), liver disease (19%), COPD (20%), and drug (14%) and alcohol (45%) dependence (Tables 2).

**Table 2.** Independent Predictors of Hospitalization Requiring Airborne, Droplet, Contact, or Other Isolation for 4,599 Patients with Positive SARS-CoV-2 Tests (2/20/2020–11/8/2020)

	Unadjusted		Age and sex		Fully adjusted	
	RR/95% CI	*p*>|*z*|	ARR/95% CI	*p*>|*z*|	ARR/95% CI	*p*>|*z*|
25-Hydroxy vitamin D, log-transformed^a^	0.96 (0.84, 1.11)	0.614	0.72 (0.63, 0.83)	<0.001	0.83 (0.72, 0.96)	0.009
Age at index date^b^, years			1.17 (1.15, 1.19)	<0.001	1.13 (1.10, 1.16)	<0.001
Male			1.30 (1.02, 1.64)	0.031	1.14 (0.90, 1.45)	0.263
Race: non-Caucasian or unknown					1.50 (1.34, 1.68)	<0.001
Ethnicity: Hispanic or Latinx					1.18 (0.98, 1.42)	0.080
Proportion of residents < federal poverty line^c^					1.03 (1.00, 1.06)	0.058
Body mass index, kg/m^2^					0.99 (0.98, 1.00)	0.049
Diabetes (any)					1.24 (1.11, 1.40)	<0.001
Cardiovascular disease (incl. hypertension)					1.27 (1.12, 1.44)	<0.001
Obstructive sleep apnea					1.12 (0.99, 1.27)	0.062
Chronic obstructive pulmonary disease					1.20 (1.06, 1.35)	0.003
Cancer					1.21 (1.08, 1.35)	0.001
Chronic kidney disease					1.44 (1.28, 1.62)	<0.001
Liver disease					1.19 (1.02, 1.40)	0.032
Human immunodeficiency virus					1.21 (0.86, 1.72)	0.276
Current or former smoker					0.99 (0.89, 1.11)	0.921
Non-alcohol drug dependence					1.14 (0.90, 1.45)	0.274
Alcohol dependence					1.45 (1.22, 1.72)	<0.001

^a^25-Hydroxy Vitamin D, log-transformed as a continuous variable, was independently associated with decreased risk of hospitalization.

^b^Relative risk reflects 5 years increase in age.

^c^Relative risk reflects 5% increase in proportion of residents in patient ZIP code living below federal poverty line.

**Table 3.** **Adjusted Risk Ratios for Hospitalization Comparing Representative 25-Hydroxy D Levels for 4,599 Patients with Positive SARS-CoV-2 Tests (2/20/2020–11/8/2020)**^a^

	Age and sex^b^ *p*<.001		Fully adjusted^c^ *P*=.009	
	ARR	95% CI	ARR	95% CI
15 ng/mL vs. 20ng/mL	1.10	(1.05, 1.14)	1.05	(1.01, 1.10)
15 ng/mL vs. 25ng/mL	1.18	(1.10, 1.27)	1.10	(1.02, 1.18)
15 ng/mL vs. 30ng/mL	1.25	(1.13, 1.38)	1.14	(1.03, 1.25)
15 ng/mL vs. 40ng/mL	1.37	(1.19, 1.58)	1.20	(1.05, 1.37)
15 ng/mL vs. 50ng/mL	1.47	(1.24, 1.75)	1.25	(1.06, 1.48)
15 ng/mL vs. 60ng/mL	1.56	(1.29, 1.90)	1.29	(1.06, 1.57)

^a^These adjusted relative risks and confidence intervals reflect comparisons between pairs of 25(OH)D values displayed in Figure 2 (fully adjusted model shown only) which were predicted from the models in Table 2.

^b^Adjusted for age and sex

^c^Adjusted for age, sex, sociodemographics (race, ethnicity, proportion of residents below federal poverty line), medical comorbidities (obesity, diabetes, cardiovascular disease including hypertension, obstructive sleep apnea, obstructive sleep apnea, chronic obstructive pulmonary disease, cancer, chronic kidney disease, liver disease, human immunodeficiency virus), and health risk behaviors (smoking status, non-alcohol drug dependence, alcohol dependence)

**Figure 2.**
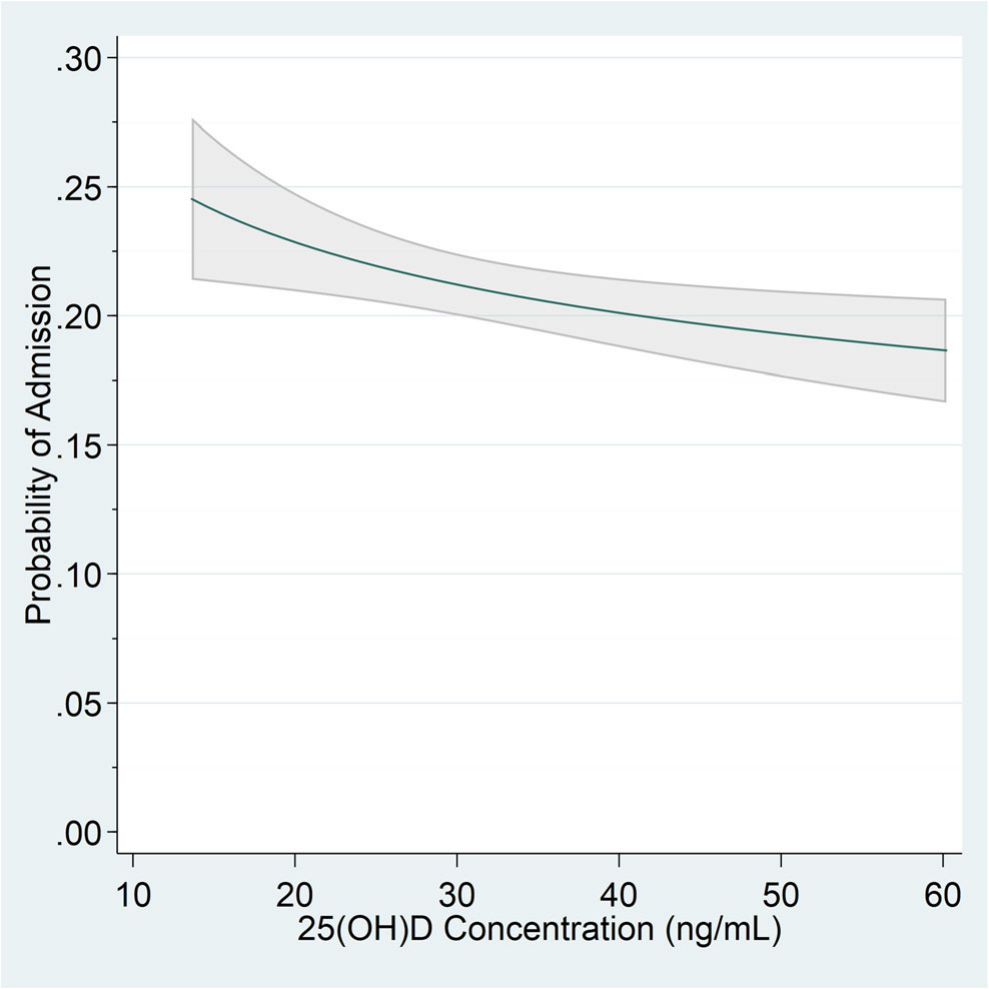
Adjusted probability of hospitalization by 25-hydroxy D concentrations for 4,599 patients with positive SARS-CoV-2 (2/20/2020–11/8/2020). Adjusted probabilities calculated as predictive margins from fully adjusted model in Table 2, which was adjusted for age, sex, sociodemographics (race, ethnicity, proportion of residents below federal poverty line), medical comorbidities (obesity, diabetes, cardiovascular disease including hypertension, obstructive sleep apnea, obstructive sleep apnea, chronic obstructive pulmonary disease, cancer, chronic kidney disease, liver disease, human immunodeficiency virus), and health risk behaviors (smoking status, non-alcohol drug dependence, alcohol dependence)

The inverse dose-response relationship between continuous increasing 25(OH)D concentrations (from 15 to 60 ng/mL) and corresponding decreasing probability of COVID-19-related hospitalization (from 24.1 to 18.7%) in fully adjusted analyses is shown in Figure 2 and Appendix Table 2. Figure 2 is non-linear demonstrating that among patients with lower 25(OH)D concentrations, increases in 25(OH)D are associated with larger reductions in probability for hospitalization than among those with higher 25(OH)D concentrations. Table 3 provides examples of representative concentrations of 25(OH)D compared to 15 ng/mL. For example, after adjusting for all other covariates, patients with a positive SARS-CoV-2 test and a 25(OH)D concentration of 15 ng/mL compared to 40 ng/mL had a 20% greater risk of hospitalization (ARR=1.20, 95% CI=1.05–1.37, *p*=0.009) (Table 3).

**Table 4.** Independent Predictors of 60-day Mortality for 4,599 Patients with Positive SARS-CoV-2 Tests (2/20/2020–11/8/2020)

	Unadjusted		Age and sex		Fully adjusted	
	RR/95% CI	*p*>|*z*|	ARR/95% CI	*p*>|*z*|	ARR/95% CI	*p*>|*z*|
25-Hydroxy vitamin D, log-transformed^a^	1.09 (0.85, 1.40)	0.495	0.61 (0.47, 0.79)	<0.001	0.65 (0.50, 0.84)	0.001
Age at index date^b^, years			1.47 (1.41, 1.52)	<0.001	1.44 (1.37, 1.51)	<0.001
Male			1.53 (0.88, 2.65)	0.133	1.28 (0.73, 2.23)	0.391
Race: non-Caucasian or unknown					1.23 (0.99, 1.53)	0.059
Ethnicity: Hispanic or Latinx					0.94 (0.63, 1.39)	0.752
Proportion of residents < federal poverty line^c^					1.00 (0.94, 1.05)	0.875
Body mass index, kg/m^2^					1.00 (0.98, 1.02)	0.979
Diabetes (any)					1.42 (1.15, 1.76)	0.001
Cardiovascular disease (incl. hypertension)					1.30 (1.03, 1.63)	0.026
Obstructive sleep apnea					1.03 (0.82, 1.29)	0.780
Chronic obstructive pulmonary Disease					1.22 (0.99, 1.50)	0.067
Cancer					1.08 (0.88, 1.33)	0.458
Chronic kidney disease					1.64 (1.33, 2.01)	<0.001
Liver disease					1.08 (0.76, 1.52)	0.669
Human immunodeficiency virus					0.63 (0.16, 2.53)	0.513
Current or former smoker					1.06 (0.86, 1.32)	0.573
Non-alcohol drug dependence					1.02 (0.57, 1.83)	0.938
Alcohol dependence					1.36 (0.91, 2.04)	0.137

^a^25-Hydroxy vitamin D, log-transformed as a continuous variable, was independently associated with decreased risk of 60-day mortality.

^b^Relative risk reflects 5 years increase in age.

^c^Relative risk reflects 5% increase in proportion of residents in patient ZIP code living below federal poverty line.

**Figure 3.**
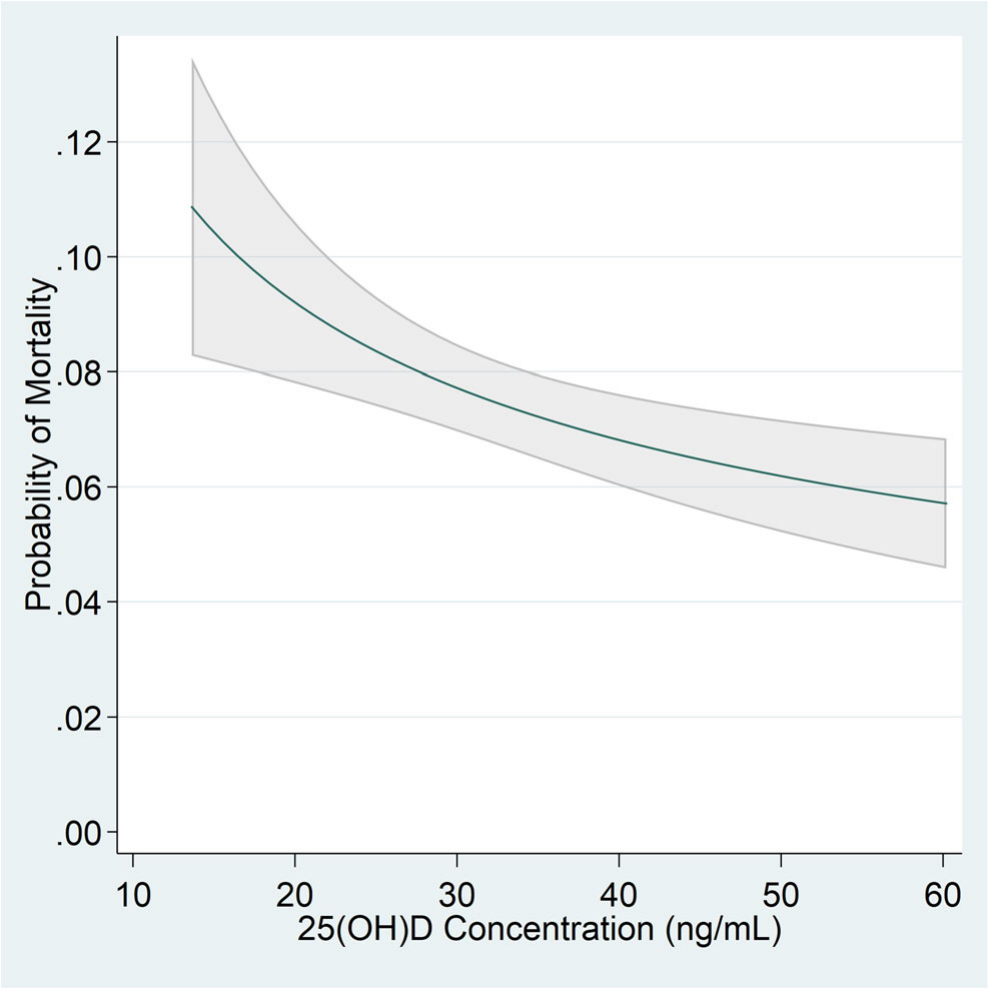
Adjusted probability of mortality by 25-hydroxy vitamin D concentrations for 4,599 patients with positive SARS-CoV-2 (2/20/2020–11/8/2020). Adjusted probabilities calculated as predictive margins from fully adjusted model in Table 4 which was adjusted for age, sex, sociodemographics (race, ethnicity, proportion of residents below federal poverty line), medical comorbidities (obesity, diabetes, cardiovascular disease including hypertension, obstructive sleep apnea, obstructive sleep apnea, chronic obstructive pulmonary disease, cancer, chronic kidney disease, liver disease, human immunodeficiency virus), and health risk behaviors (smoking status, non-alcohol drug dependence, alcohol dependence)

Of the 4,599 patients with positive SARS-CoV-2 tests, 340 (7.4%) died within 60 days of their index SARS-CoV-2 test. Of those who died, 48 (14.1%) had vitamin D concentrations <20 ng/mL. Increasing 25(OH)D concentrations were independently associated with decreasing risk of COVID-19-related mortality after adjusting for all covariates (*p*=.001) (Table 4). Other risk factors significantly associated with increased risk for COVID-19-related mortality were age (44% per 5-year increase), diabetes (42%), CVD including hypertension (30%), and chronic kidney disease (64% increase) (Table 4). The inverse dose-response relationship between continuous increasing 25(OH)D concentrations (from 15 to 60 ng/mL) and decreasing probability of COVID-19-related mortality within 60 days (from 10.4 to 5.7%, *p*=0.001) is illustrated in Figure 3 and Appendix Table 3. Figure 3 is non-linear demonstrating that among patients with the lower 25(OH)D concentrations, increases in 25(OH)D are associated with larger reductions in mortality than among those with higher 25(OH)D concentrations. Table 5 provides examples of representative concentrations of 25(OH)D compared to 15 ng/mL. For example, in a fully adjusted model, patients with positive SARS-CoV-2 tests and 25(OH)D concentrations of 15ng/mL compared with 40ng/mL had an increased risk of mortality of 53% (ARR=1.53; 95% CI=1.18–1.98, *p*=0.001) (Table 5).

**Table 5.** **Risk Ratio for Mortality Comparing Representative 25-Hydroxy D Concentrations for 4,599 Patients with Positive SARS-CoV-2 Tests (2/20/2020–11/8/2020)**^a^

	Age and sex^b^ *P*<.001		Fully adjusted^c^ *P*=.001	
	ARR	95% CI	ARR	95% CI
15 ng/mL vs. 20ng/mL	1.15	(1.07, 1.24)	1.13	(1.05, 1.22)
15 ng/mL vs. 25ng/mL	1.29	(1.13, 1.47)	1.25	(1.09, 1.43)
15 ng/mL vs. 30ng/mL	1.41	(1.18, 1.69)	1.35	(1.13, 1.62)
15 ng/mL vs. 40ng/mL	1.63	(1.26, 2.10)	1.53	(1.18, 1.98)
15 ng/mL vs. 50ng/mL	1.82	(1.32, 2.49)	1.68	(1.23, 2.31)
15 ng/mL vs. 60ng/mL	1.99	(1.38, 2.86)	1.82	(1.27, 2.63)

^a^These adjusted relative risks and confidence intervals reflect comparisons between pairs of 25(OH)D values displayed in Figure 3 (fully adjusted model shown only) which were predicted from the models in Table 4.

^b^Adjusted for age and sex

^c^Adjusted for age, sex, sociodemographics (race, ethnicity, proportion of residents below federal poverty line), medical comorbidities (obesity, diabetes, cardiovascular disease including hypertension, obstructive sleep apnea, obstructive sleep apnea, chronic obstructive pulmonary disease, cancer, chronic kidney disease, liver disease, human immunodeficiency virus), and health risk behaviors (smoking status, non-alcohol drug dependence, alcohol dependence)

## DISCUSSION

To our knowledge, this is the first observational study to demonstrate a dose-response relationship between the exposure — lower concentrations of 25(OH)D — and the outcome — increased COVID-19-related hospitalization and mortality — in a large geographically and racially and ethnically diverse cohort of VA patients testing positive for SARS-CoV-2. Furthermore, this relationship was non-linear. Patients with the lowest 25(OH)D concentrations had the greatest associated reduction in hospitalization and mortality as 25(OH)D concentrations increased. These findings remained significant even after adjusting for known risk factors for both vitamin D deficiency and COVID-19 severity. These findings contribute to a growing evidence-base suggesting that vitamin D deficiency may be associated with more severe outcomes related to COVID-19.^19,21–25^ Indeed, pre-clinical studies demonstrate that 25(OH)D stimulates immune and respiratory epithelial cells to secrete cathelicidin, an anti-microbial peptide that clears respiratory pathogens. 25(OH)D also initiates adaptive immunity to dampen down pro-inflammatory cytokines (“cytokine storm”) leading to adverse COVID-19 outcomes.^45,46^

This study found that Black or African American patients had over twice the risk for low 25(OH)D concentrations consistent with 25(OH)D deficiency. In Black and other non-White individuals, darker skin pigmentation blocks ultraviolet light; thus, more solar radiation may be required to produce similar concentrations of 25(OH)D, although the etiology of low 25(OH)D in non-White individuals remains controversial.^12,15,47,48^ In addition, stay-at-home orders or quarantining related to COVID-19 may have resulted in even less sun exposure, putting some individuals at greater risk for vitamin D deficiency.^49^ Furthermore, ad hoc analyses of the larger cohort of 71,175 with positive SARS-CoV-2 tests (Figure 1) revealed that patients who were Caucasian, older, female, and had a variety of chronic health conditions including CVD, COPD, and cancer were significantly more likely to have been tested for 25(OH)D in the 15–90 days prior to their index positive SARS-CoV-2 test (Appendix Table 4). This may explain why some patients in this cohort with chronic conditions were less likely to be vitamin D deficient — they had been tested and likely supplemented with vitamin D (a finding confirmed by additional ad hoc analyses). In contrast, Black or African American patients were significantly less likely than Caucasian patients to have been tested for 25(OH)D within the VA in the past year (Appendix Table 4), hence less likely that vitamin D deficiency would have been detected or treated. This is consistent with studies revealing disparities in access to care within VA among ethnic/racial minority groups, which may also explain the lower 25(OH)D concentrations observed in non-White populations.^50,51^

Black or African American populations have been disproportionately impacted by the COVID-19 pandemic through higher infection rates and more serious clinical outcomes, thought to be related to increased exposure, poorer access to health care, and to a lesser extent, underlying pre-existing conditions.^9,52^ Latinx patients and patients residing in areas with higher levels of poverty were also at significantly greater risk for vitamin D deficiency. In addition, patients with drug and alcohol dependence were more likely to have low 25(OH)D concentrations, and those with alcohol dependence to be hospitalized for COVID-19-related complications. Individuals with substance use disorders, as well as those living in poverty, may have lower intake of vitamin D enriched foods or supplements, coupled with a lack of or increased requirement for sunlight to produce 25(OH)D. Thus, testing for 25(OH)D and supplementing when indicated, especially in non-Whiteracial/ethnic groups, poor individuals, and those with substance use disorders, may represent one way to mitigate social disparities associated with poorer COVID-19-related clinical outcomes.^40,52^

Vitamin D deficiency was strongly associated with mortality related to COVID-19 infection. Steadily decreasing concentrations of vitamin D from 60 to 15 ng/mL corresponded to a continuous and significant increase in mortality in patients hospitalized for COVID-19 infection after adjustment for sociodemographics, including race/ethnicity and medical comorbidities. Only a handful of other smaller studies have demonstrated this association between vitamin D deficiency and COVID-19-related deaths;^22–24^ thus, more research is needed to rule out the possibility that 25(OH)D is instead serving as a marker for other unmeasured predictors of mortality in patients with COVID-19. Nevertheless, the non-linear dose-response relationship between 25(OH)D concentrations and risk of both COVID-19-related hospitalization and mortality provides tentative support for providing vitamin D supplementation.

This analysis has limitations. First, results are based on VA administrative data that incompletely capture all possible risk factors for COVID-19 hospitalization and mortality, in addition to missing, unknown, or misclassified race/ethnicity data and a lack of income and educational data.^53,54^ Second, the retrospective cohort of US veterans was not representative of all veterans or the US population; it was also largely male, and thus our results may not completely generalize. Third, results may be biased as to which VA patients are tested for 25(OH)D; as ad hoc analyses revealed, those with 25(OH)D test results were more likely to be White, older, and sicker. Thus, while we adjusted for these covariates, there may be additional unmeasured factors associated with being tested for 25(OH)D. Fourth, while we excluded patients with 25(OH)D results within 14 days of a positive SARS-CoV-2 test, we cannot be sure that 25(OH)D concentrations measured within 15 to 90 days of the positive test did not act as a negative acute phase reactant, even outside the prodromal or infectious period.^33^ Finally, we were unable to reliably investigate the role of vitamin D supplementation since VA pharmacy data only capture vitamin D dispensed through VA pharmacies.

Considering these limitations, our findings suggest that lower 25(OH)D concentrations have a significant and independent dose-response relationship with adverse clinical outcomes of COVID-19 infection, namely hospitalization and mortality, as it did in this large, diverse cohort of VA patients tested both for COVID-19 and 25(OH)D. Compared to other COVID-19 therapies, vitamin D supplementation is relatively inexpensive, widely available, and safe for most individuals at therapeutic doses. Therefore, consideration should be given to testing patients for 25(OH)D who are SARS-CoV-2 positive or at high risk for COVID-19-related complications and providing vitamin D supplementation when deficient. Only a large randomized controlled trial can confirm if vitamin D supplementation can prevent hospitalization and mortality in patients with COVID-19. Another consideration brought to light by this study is that testing for and supplementing those with low 25(OH) D concentrations may differentially benefit vulnerable sub-populations of patients disproportionately negatively impacted by COVID-19.

## Supplementary Information

ESM 1 (DOCX 69 kb)

## References

[1] United Nations Development Program (UNDP). Coronavirus disease COVID-19 pandemic

[2] Gerberding JL (2020). Measuring Pandemic Impact: Vital Signs From Vital Statistics. Ann Intern Med..

[3] **Thompson, MG, Burgess JL, Naleway AL**, al. e. Interim Estimates of Vaccine Effectiveness of BNT162b2 and mRNA-1273 COVID-19 Vaccines in Preventing SARS-CoV-2 Infection Among Health Care Personnel, First Responders, and Other Essential and Frontline Workers — Eight U.S. Locations, December 2020–March 2021. *MMWR Morb Mortal Wkly Rep 2021.* 2021;70:495-500. 10.15585/mmwr.mm7013e3 PMC8022879 33793460

[4] **McCabe SD, Hammershaimb EA, Cheng D, et al.** Unraveling Attributes of COVID-19 Vaccine Hesitancy in the U.S.: A Large Nationwide Study. *medRxiv.* 2021. 10.1038/s41598-023-34340-3 PMC10209066 37225748

[5] Emanuel EJ, Luna F, Schaefer GO, Tan KC, Wolff J (2021). Enhancing the WHO’s Proposed Framework for Distributing COVID-19 Vaccines Among Countries. Am J Public Health..

[6] Emanuel EJ, Persad G, Kern A (2020). An ethical framework for global vaccine allocation. Science..

[7] **Thebault R**. Are we entering a ‘fourth wave’ of the pandemic? Experts disagree. *Washington Post*2021.

[8] Mackey K, Ayers CK, Kondo KK (2021). Racial and Ethnic Disparities in COVID-19-Related Infections, Hospitalizations, and Deaths : A Systematic Review. Ann Intern Med..

[9] Webb Hooper M, Napoles AM, Perez-Stable EJ (2020). COVID-19 and Racial/Ethnic Disparities. JAMA..

[10] Dawson-Hughes B, Mithal A, Bonjour JP (2010). IOF position statement: vitamin D recommendations for older adults. Osteoporos Int..

[11] Institute of Medicine. *Dietary Reference Intakes for Calcium and Vitamin D.* Washington, D.C.2011.

[12] Mithal A, Wahl DA, Bonjour JP (2009). Global vitamin D status and determinants of hypovitaminosis D. Osteoporos Int..

[13] Palacios C, Gonzalez L (2014). Is vitamin D deficiency a major global public health problem?. J Steroid Biochem Mol Biol.

[14] MacLaughlin J, Holick MF (1985). Aging decreases the capacity of human skin to produce vitamin D3. J Clin Invest..

[15] Forrest KY, Stuhldreher WL (2011). Prevalence and correlates of vitamin D deficiency in US adults. Nutr Res..

[16] Yetley EA (2008). Assessing the vitamin D status of the US population. Am J Clin Nutr..

[17] Webb AR, Kline L, Holick MF (1988). Influence of season and latitude on the cutaneous synthesis of vitamin D3: exposure to winter sunlight in Boston and Edmonton will not promote vitamin D3 synthesis in human skin. J Clin Endocrinol Metab..

[18] Meltzer DO, Best TJ, Zhang H, Vokes T, Arora V, Solway J (2020). Association of Vitamin D Status and Other Clinical Characteristics With COVID-19 Test Results. JAMA Netw Open..

[19] Merzon E, Tworowski D, Gorohovski A (2020). Low plasma 25(OH) vitamin D level is associated with increased risk of COVID-19 infection: an Israeli population-based study. FEBS J..

[20] Kaufman HW, Niles JK, Kroll MH, Bi C, Holick MF (2020). SARS-CoV-2 positivity rates associated with circulating 25-hydroxyvitamin D levels. PLoS One..

[21] De Smet D, De Smet K, Herroelen P, Gryspeerdt S, Martens GA (2021). Serum 25(OH)D Level on Hospital Admission Associated With COVID-19 Stage and Mortality. Am J Clin Pathol..

[22] Karahan S, Katkat F (2021). Impact of Serum 25(OH) Vitamin D Level on Mortality in Patients with COVID-19 in Turkey. J Nutr Health Aging..

[23] Angelidi AM, Belanger MJ, Lorinsky MK (2021). Vitamin D Status Is Associated With In-Hospital Mortality and Mechanical Ventilation: A Cohort of COVID-19 Hospitalized Patients. Mayo Clin Proc..

[24] Entrenas Castillo M, Entrenas Costa LM, Vaquero Barrios JM (2020). Effect of calcifediol treatment and best available therapy versus best available therapy on intensive care unit admission and mortality among patients hospitalized for COVID-19: A pilot randomized clinical study. J Steroid Biochem Mol Biol..

[25] **Rastogi A, Bhansali A, Khare N, et al.** Short term, high-dose vitamin D supplementation for COVID-19 disease: a randomised, placebo-controlled, study (SHADE study). *Postgrad Med J.* 2020. 10.1136/postgradmedj-2020-139065 33184146

[26] Murai IH, Fernandes AL, Sales LP (2021). Effect of a Single High Dose of Vitamin D3 on Hospital Length of Stay in Patients With Moderate to Severe COVID-19: A Randomized Clinical Trial. JAMA..

[27] **Pittas AG, Jorde R, Kawahara T, Dawson-Hughes B**. Vitamin D Supplementation for Prevention of Type 2 Diabetes Mellitus: To D or Not to D? *J Clin Endocrinol Metab.* 2020;105(12). 10.1210/clinem/dgaa594 PMC7571449 32844212

[28] Zittermann A, Pilz S (2019). Vitamin D and Cardiovascular Disease: An Update. Anticancer Res..

[29] **Bikle DD**. Vitamin D and cancer: the promise not yet fulfilled. *Endocrine.* 2014;46(1):29-38. 10.1007/s12020-013-0146-1 PMC3976762 24402695

[30] **Boccardi V, Lapenna M, Gaggi L, et al.** Hypovitaminosis D: A Disease Marker in Hospitalized Very Old Persons at Risk of Malnutrition. *Nutrients.* 2019;11(1). 10.3390/nu11010128 PMC6357065 30634546

[31] Gandhi RT, Lynch JB, Del Rio C (2020). Mild or Moderate Covid-19. N Engl J Med..

[32] National Institutes of Health. COVID-19 Treatment Guidelines: Vitamin D. In:July 17, 2020. 34003615

[33] Waldron JL, Ashby HL, Cornes MP (2013). Vitamin D: a negative acute phase reactant. J Clin Pathol..

[34] *SAS System* [computer program]. Version 9.4. Cary, North Carolina, U.S.A.: SAS Institute; 2013.

[35] *Stata: Release 15 Statistical Software.* [computer program]. College Station, Texas, U.S.A.: StataCorp LLC; 2017.

[36] Thienpont LM, Stepman HC, Vesper HW (2012). Standardization of measurements of 25-hydroxyvitamin D3 and D2. Scand J Clin Lab Invest Suppl..

[37] Binkley N, Carter GD (2017). Toward Clarity in Clinical Vitamin D Status Assessment: 25(OH)D Assay Standardization. Endocrinol Metab Clin North Am..

[38] Holick MF, Binkley NC, Bischoff-Ferrari HA (2011). Evaluation, treatment, and prevention of vitamin D deficiency: an Endocrine Society clinical practice guideline. J Clin Endocrinol Metab..

[39] U.S. Census Bureau. American Community Survey. *American Community Survey 5-Year Estimates. Table 1901. .* 2018.

[40] Burstrom B, Tao W (2020). Social determinants of health and inequalities in COVID-19. Eur J Public Health..

[41] https://www.research.va.gov/resources/CIPHER.pdf. Accessed.

[42] Zou G (2004). A modified poisson regression approach to prospective studies with binary data. Am J Epidemiol..

[43] **Bikle DD, Schwartz J.** Vitamin D Binding Protein, Total and Free Vitamin D Levels in Different Physiological and Pathophysiological Conditions. *Frontiers in Endocrinology.* 2019;10(317). 10.3389/fendo.2019.00317 PMC6546814 31191450

[44] Cummings P (2011). Estimating Adjusted Risk Ratios for Matched and Unmatched Data: An Update. The Stata Journal..

[45] Bilezikian JP, Bikle D, Hewison M (2020). MECHANISMS IN ENDOCRINOLOGY: Vitamin D and COVID-19. Eur J Endocrinol..

[46] Mehta P, McAuley DF, Brown M (2020). COVID-19: consider cytokine storm syndromes and immunosuppression. Lancet..

[47] **Rhodes JM, Subramanian S, Laird E, Griffin G, Kenny RA**. Perspective: Vitamin D deficiency and COVID-19 severity - plausibly linked by latitude, ethnicity, impacts on cytokines, ACE2 and thrombosis. *J Intern Med.* 2020. 10.1111/joim.13149 PMC7361294 32613681

[48] Young AR, Morgan KA, Ho TW (2020). Melanin has a Small Inhibitory Effect on Cutaneous Vitamin D Synthesis: A Comparison of Extreme Phenotypes. J Invest Dermatol..

[49] DeLuccia R, Clegg D, Sukumar D (2021). The implications of vitamin D deficiency on COVID-19 for at-risk populations. Nutr Rev..

[50] Saha S, Freeman M, Toure J, Tippens KM, Weeks C, Ibrahim S (2008). Racial and ethnic disparities in the VA health care system: a systematic review. J Gen Intern Med..

[51] Peterson K, Anderson J, Boundy E, Ferguson L, McCleery E, Waldrip K (2018). Mortality Disparities in Racial/Ethnic Minority Groups in the Veterans Health Administration: An Evidence Review and Map. Am J Public Health..

[52] **Lopez L, Hart LH, Katz MH**. Racial and Ethnic Health Disparities Related to COVID-19. *JAMA.* 2021. 10.1001/jama.2020.26443 33480972

[53] Kressin NR, Chang BH, Hendricks A, Kazis LE (2003). Agreement between administrative data and patients’ self-reports of race/ethnicity. Am J Public Health..

[54] Hamilton NS, Edelman D, Weinberger M, Jackson GL (2009). Concordance between self-reportedrace/ethnicity and that recorded in a Veteran Affairs electronic medical record. N C Med J..

