# Supplementary material for: Association of Vitamin D Status and COVID-19-Related Hospitalization and Mortality
Source: J Gen Intern Med. 2022 Jan 1;37(4):853–61. doi: 10.1007/s11606-021-07170-0 (PMC8723909; doi:10.1007/s11606-021-07170-0)
Supplement: Supplementary file 1 — (DOCX 69 kb) [file 11606_2021_7170_MOESM1_ESM.docx]

**Appendix Table 1. Additional Information on 25(OH)D Assays Used by VA Clinical Laboratories Included in this Study**

| 25(OH)D Assays and Results Included in Study | | | 25(OH)D Assay Performance Comparison on 2020 CAP PT Survey^b^ | | | |
| --- | --- | --- | --- | --- | --- | --- |
| 25(OH)D Assays Used by VA Laboratories | Instruments Used | Results Included (% of total) | Mean (ng/mL) of CAP PT Sample VITD-01 | S.D./C.V. (%) for  Sample VITD-01 | Mean (ng/mL) of CAP PT Sample VITD-04 | S.D./C.V. (%) for  Sample VITD-04 |
| Abbott | Architect | 39% | 17.17 | 1.0/5.8 | 48.14 | 2.19/4.5 |
| Siemens | Advia Centaur | 24% | 15.34 | 2.34/15.2 | 40.77 | 4.41/10.8 |
| Beckman | DxI | 22% | 17.49 | 2.48/14.2 | 50.45 | 4.74/9.4 |
| Ortho | Vitros | 5.40% | 17.38 | 2.54/14.6 | 53.1 | 4.62/8.7 |
| DiaSorin | Liaison | 3.60% | 16.88 | 1.44/8.5 | 44.22 | 3.39/7.7 |
| Roche | Cobas | 3.30% | 17.47 | 1.49/8.5 | 52.32 | 4.45/8.5 |
| IDS | IDS-iSYS | 2.70% | N/A^c^ | N/A^c^ | N/A^c^ | N/A^c^ |
|  | LC-MS/MS^a^ | N/A | 18.89 | 0.87/4.6 | 46.18 | 2.93/6.3 |

1. No VA laboratory uses LC-MS/MS, but LC-MS/MS is included in College of Am Pathologists (CAP) proficiency testing (PT) program. Listed here as reference method.
2. Data from CAP PT survey Participant Summary Reports (25-OH Vitamin D: VITD-A 2020 & VITD-B 2020), which shows that results of the 25(OH)D assays are comparable at "low" (VITD-01) and "high" (VITD-04) levels without clinically significant biases.
3. No data entry because too few peer group laboratories use IDS 25(OH)D assay.

*C.V.: coefficient of variation (%); N/A: not applicable; S.D.: standard deviation; VITD: vitamin D test survey sample; 25(OH)D: 25-hydroxyvitamin D.*

**Appendix Table 2. Probabilities for Hospitalization for Representative Blood 25(OH)D Concentrations for 4,599 Patients with Positive SARS-CoV-2 Tests (2/20/2020 – 11/8/2020)**

|  | Age and Sex Adjusted*^a^* | | Fully Adjusted*^b^* | |
| --- | --- | --- | --- | --- |
|  | Probability | (95% CI) | Probability | (95% CI) |
| 15ng/ml | 0.268 | (0.237, 0.300) | 0.241 | (0.213, 0.269) |
| 20ng/ml | 0.245 | (0.224, 0.266) | 0.229 | (0.209, 0.248) |
| 25ng/ml | 0.228 | (0.213, 0.243) | 0.219 | (0.205, 0.233) |
| 30ng/ml | 0.215 | (0.202, 0.227) | 0.212 | (0.200, 0.224) |
| 40ng/ml | 0.196 | (0.182, 0.209) | 0.201 | (0.188, 0.214) |
| 50ng/ml | 0.182 | (0.166, 0.199) | 0.193 | (0.176, 0.210) |
| 60ng/ml | 0.172 | (0.153, 0.191) | 0.187 | (0.167, 0.207) |

1. Adjusted probabilities calculated as predictive margins from model in Table 2, which was adjusted for age and sex
2. Adjusted probabilities calculated as predictive margins from model in Table 2, which was adjusted for age, sex, sociodemographics (race, ethnicity, proportion of residents below federal poverty line), medical comorbidities (obesity, diabetes, cardiovascular disease including hypertension, obstructive sleep apnea, obstructive sleep apnea, chronic obstructive pulmonary disease, cancer, chronic kidney disease, liver disease, Human Immunodeficiency Virus), and health risk behaviors (smoking status, non-alcohol drug dependence, alcohol dependence)

**Appendix Table 3. Probabilities for Mortality for Representative Blood 25(OH)D Concentrations for 4,599 Patients with Positive SARS-CoV-2 Tests (2/20/2020 – 11/8/2020)**

|  | Age and Sex Adjusted *^a^* | | Fully Adjusted*^b^* | |
| --- | --- | --- | --- | --- |
|  | Probability | (95% CI) | Probability | (95% CI) |
| 15ng/ml | 0.110 | (0.086, 0.133) | 0.104 | (0.082, 0.127) |
| 20ng/ml | 0.095 | (0.081, 0.110) | 0.092 | (0.078, 0.106) |
| 25ng/ml | 0.085 | (0.076, 0.095) | 0.084 | (0.074, 0.093) |
| 30ng/ml | 0.078 | (0.070, 0.086) | 0.077 | (0.070, 0.085) |
| 40ng/ml | 0.068 | (0.060, 0.075) | 0.068 | (0.060, 0.076) |
| 50ng/ml | 0.060 | (0.051, 0.070) | 0.062 | (0.052, 0.072) |
| 60ng/ml | 0.055 | (0.044, 0.066) | 0.057 | (0.046, 0.068) |

1. Adjusted probabilities calculated as predictive margins from model in Table 3, which was adjusted for age and sex
2. Adjusted probabilities calculated as predictive margins from model in Table 3, which was adjusted for age, sex, sociodemographics (race, ethnicity, proportion of residents below federal poverty line), medical comorbidities (obesity, diabetes, cardiovascular disease including hypertension, obstructive sleep apnea, obstructive sleep apnea, chronic obstructive pulmonary disease, cancer, chronic kidney disease, liver disease, Human Immunodeficiency Virus), and health risk behaviors (smoking status, non-alcohol drug dependence, alcohol dependence)

**Appendix Table 4. Factors Associated with Having Been Tested for 25(OH)D during the 15 to 90 Days Preceding the Index SARS-CoV-2 Positive Test**

|  | All  Patients |  |  | No 25(OH)D Results Available | |  | 1 or More 25(OH)D Results Available | |  |  |  |
| --- | --- | --- | --- | --- | --- | --- | --- | --- | --- | --- | --- |
|  |  |  |  | N=66,303 (%) | |  | N=4,872 (%) | |  | Relative Risk (95% CI) | Pr>\|Z\| |
| **Sociodemographic Factors** |  |  |  |  |  |  |  |  |  |  |  |
| Age, mean (SD), y*^a^* | 60.2 | (16.6) |  | 60.1 | (16.7) |  | 62.5 | (15.2) |  | 1.04 (1.03, 1.05) | <0.001 |
| Sex |  |  |  |  |  |  |  |  |  |  |  |
| Male | 63,652 | 89.4% |  | 59,399 | 89.6% |  | 4,253 | 87.3% |  |  |  |
| Female | 7,523 | 10.6% |  | 6,904 | 10.4% |  | 619 | 12.7% |  | 1.23 (1.14, 1.34) | <0.001 |
| Race |  |  |  |  |  |  |  |  |  |  |  |
| White | 44,168 | 62.1% |  | 40,961 | 61.8% |  | 3,207 | 65.8% |  |  |  |
| Black or African American | 19,584 | 27.5% |  | 18,359 | 27.7% |  | 1,225 | 25.1% |  | 0.86 (0.81, 0.92) | <0.001 |
| American Indian or Alaska Native | 694 | 1.0% |  | 653 | 1.0% |  | 41 | 0.8% |  | 0.81 (0.60, 1.10) | 0.177 |
| Asian | 614 | 0.9% |  | 571 | 0.9% |  | 43 | 0.9% |  | 0.96 (0.72, 1.29) | 0.810 |
| Native Hawaiian or Pacific Islander | 671 | 0.9% |  | 630 | 1.0% |  | 41 | 0.8% |  | 0.84 (0.62, 1.13) | 0.258 |
| Unknown | 5,444 | 7.6% |  | 5,129 | 7.7% |  | 315 | 6.5% |  | 0.80 (0.71, 0.89) | <0.001 |
| Ethnicity |  |  |  |  |  |  |  |  |  |  |  |
| Not Hispanic or Latinx | 63,075 | 88.6% |  | 58,724 | 88.6% |  | 4,351 | 89.3% |  |  |  |
| Hispanic Or Latinx | 8,100 | 11.4% |  | 7,579 | 11.4% |  | 521 | 10.7% |  | 0.93 (0.85, 1.02) | 0.119 |
| Percent of Residents < Federal Poverty Line, mean (SD) *^b^* | 15.9 | (9.4) |  | 15.9 | (9.4) |  | 16.2 | (9.2) |  | 1.01 (1.00, 1.03) | 0.126 |
| **Medical Comorbidities** |  |  |  |  |  |  |  |  |  |  |  |
| Body Mass Index |  |  |  |  |  |  |  |  |  |  |  |
| BMI Under 18 | 564 | 0.8% |  | 515 | 0.8% |  | 49 | 1.0% |  |  |  |
| BMI 18-24 | 10,891 | 15.5% |  | 10,147 | 15.5% |  | 744 | 15.4% |  | 0.79 (0.60, 1.04) | 0.087 |
| BMI 25-29 | 22,616 | 32.2% |  | 21,100 | 32.3% |  | 1,516 | 31.3% |  | 0.77 (0.59, 1.01) | 0.061 |
| BMI 30-34 | 20,141 | 28.7% |  | 18,752 | 28.7% |  | 1,389 | 28.7% |  | 0.79 (0.60, 1.04) | 0.097 |
| BMI 35+ | 15,971 | 22.8% |  | 14,825 | 22.7% |  | 1,146 | 23.7% |  | 0.83 (0.63, 1.09) | 0.171 |
| Low Serum Albumin |  |  |  |  |  |  |  |  |  |  |  |
| Normal Serum Albumin | 69,358 | 97.4% |  | 65,057 | 98.1% |  | 4,301 | 88.3% |  |  |  |
| Low Serum Albumin | 1,817 | 2.6% |  | 1,246 | 1.9% |  | 571 | 11.7% |  | 5.07 (4.71, 5.46) | <0.001 |
| Diabetes (Any Type) |  |  |  |  |  |  |  |  |  |  |  |
| No | 46,030 | 64.7% |  | 43,297 | 65.3% |  | 2,733 | 56.1% |  |  |  |
| Yes | 25,145 | 35.3% |  | 23,006 | 34.7% |  | 2,139 | 43.9% |  | 1.43 (1.36, 1.51) | <0.001 |
| Cardiovascular Dis. (incl. Hypertension) |  |  |  |  |  |  |  |  |  |  |  |
| No | 47,894 | 67.3% |  | 45,001 | 67.9% |  | 2,893 | 59.4% |  |  |  |
| Yes | 23,281 | 32.7% |  | 21,302 | 32.1% |  | 1,979 | 40.6% |  | 1.41 (1.33, 1.49) | <0.001 |
| Obstructive Sleep Apnea |  |  |  |  |  |  |  |  |  |  |  |
| No | 48,754 | 68.5% |  | 45,592 | 68.8% |  | 3,162 | 64.9% |  |  |  |
| Yes | 22,421 | 31.5% |  | 20,711 | 31.2% |  | 1,710 | 35.1% |  | 1.18 (1.11, 1.24) | <0.001 |
| Chronic Obstructive Pulmonary Disease |  |  |  |  |  |  |  |  |  |  |  |
| No | 60,041 | 84.4% |  | 56,194 | 84.8% |  | 3,847 | 79.0% |  |  |  |
| Yes | 11,134 | 15.6% |  | 10,109 | 15.2% |  | 1,025 | 21.0% |  | 1.44 (1.35, 1.53) | <0.001 |
| Cancer |  |  |  |  |  |  |  |  |  |  |  |
| No | 57,228 | 80.4% |  | 53,565 | 80.8% |  | 3,663 | 75.2% |  |  |  |
| Yes | 13,947 | 19.6% |  | 12,738 | 19.2% |  | 1,209 | 24.8% |  | 1.35 (1.27, 1.44) | <0.001 |
| Chronic Kidney Disease |  |  |  |  |  |  |  |  |  |  |  |
| No | 61,195 | 86.0% |  | 57,345 | 86.5% |  | 3,850 | 79.0% |  |  |  |
| Yes | 9,980 | 14.0% |  | 8,958 | 13.5% |  | 1,022 | 21.0% |  | 1.63 (1.52, 1.74) | <0.001 |
| Liver Disease |  |  |  |  |  |  |  |  |  |  |  |
| No | 66,546 | 93.5% |  | 62,084 | 93.6% |  | 4,462 | 91.6% |  |  |  |
| Yes | 4,629 | 6.5% |  | 4,219 | 6.4% |  | 410 | 8.4% |  | 1.32 (1.20, 1.46) | <0.001 |
| Human Immunodeficiency Virus |  |  |  |  |  |  |  |  |  |  |  |
| No | 70,480 | 99.0% |  | 65,668 | 99.0% |  | 4,812 | 98.8% |  |  |  |
| Yes | 695 | 1.0% |  | 635 | 1.0% |  | 60 | 1.2% |  | 1.26 (0.99, 1.61) | 0.059 |
| **Health Risk Behaviors** |  |  |  |  |  |  |  |  |  |  |  |
| Smoking Status |  |  |  |  |  |  |  |  |  |  |  |
| Never Smoker | 28,392 | 39.9% |  | 26,389 | 39.8% |  | 2,003 | 41.1% |  |  |  |
| Current or Former Smoker | 42,783 | 60.1% |  | 39,914 | 60.2% |  | 2,869 | 58.9% |  | 0.95 (0.90, 1.00) | 0.072 |
| Non-Alcohol Drug Dependence |  |  |  |  |  |  |  |  |  |  |  |
| No | 68,141 | 95.7% |  | 63,491 | 95.8% |  | 4,650 | 95.4% |  |  |  |
| Yes | 3,034 | 4.3% |  | 2,812 | 4.2% |  | 222 | 4.6% |  | 1.07 (0.94, 1.22) | 0.289 |
| Alcohol Dependence |  |  |  |  |  |  |  |  |  |  |  |
| No | 64,135 | 90.1% |  | 59,747 | 90.1% |  | 4,388 | 90.1% |  |  |  |
| Yes | 7,040 | 9.9% |  | 6,556 | 9.9% |  | 484 | 9.9% |  | 1.00 (0.92, 1.10) | 0.920 |

a. Relative risk reflects 5 years increase in age.

b. U.S. Census Bureau. American Community Survey, 2018 American Community Survey 5-Year Estimates. Table 1901. Accessed October 2, 2020. https://data.census.gov/cedsci/. Relative risk reflects 5% increase in proportion of residents in patient ZIP code living below federal poverty line.

**Appendix Table 5. Sensitivity Analysis – Including Serum Albumin with Independent Predictors of Hospitalization Requiring Airborne, Droplet, Contact, or Other Isolation for 4,599 Patients with Positive SARS-CoV-2 Tests (2/20/2020 – 11/8/2020)**

|  | Unadjusted |  | Age and Sex |  | Fully Adjusted |  |
| --- | --- | --- | --- | --- | --- | --- |
|  | RR/95% CI | p>\|z\| | ARR/95% CI | p>\|z\| | ARR/95% CI | p>\|z\| |
| 25-Hydroxy Vitamin D, log-transformed ^a^ | 0.96 (0.84,1.11) | 0.614 | 0.72 (0.63,0.83) | <0.001 | 0.87 (0.75,1.00) | 0.043 |
| Age at Index Date *^b^*, years |  |  | 1.17 (1.15,1.19) | <0.001 | 1.12 (1.10,1.15) | <0.001 |
| Male |  |  | 1.30 (1.02,1.64) | 0.031 | 1.16 (0.91,1.47) | 0.222 |
| **Low Serum Albumin** |  |  |  |  | **1.41 (1.24,1.61)** | **<0.001** |
| Race: Non-Caucasian or Unknown |  |  |  |  | 1.50 (1.34,1.68) | <0.001 |
| Ethnicity: Hispanic or Latinx |  |  |  |  | 1.20 (1.00,1.44) | 0.051 |
| Proportion of Residents < Federal Poverty Line *^c^* |  |  |  |  | 1.03 (1.00,1.06) | 0.058 |
| Body Mass Index, kg/m^2^ |  |  |  |  | 0.99 (0.98,1.00) | 0.060 |
| Diabetes (Any) |  |  |  |  | 1.23 (1.09,1.39) | 0.001 |
| Cardiovascular Disease (incl. Hypertension) |  |  |  |  | 1.24 (1.09,1.41) | 0.001 |
| Obstructive Sleep Apnea |  |  |  |  | 1.13 (1.00,1.27) | 0.055 |
| Chronic Obstructive Pulmonary Disease |  |  |  |  | 1.17 (1.04,1.32) | 0.011 |
| Cancer |  |  |  |  | 1.19 (1.06,1.33) | 0.002 |
| Chronic Kidney Disease |  |  |  |  | 1.38 (1.23,1.56) | <0.001 |
| Liver Disease |  |  |  |  | 1.16 (0.99,1.36) | 0.068 |
| Human Immunodeficiency Virus |  |  |  |  | 1.24 (0.88,1.75) | 0.217 |
| Current or Former Smoker |  |  |  |  | 0.99 (0.88,1.11) | 0.858 |
| Non-Alcohol Drug Dependence |  |  |  |  | 1.12 (0.88,1.42) | 0.350 |
| Alcohol Dependence |  |  |  |  | 1.40 (1.18,1.67) | <0.001 |

1. 25-Hydroxy Vitamin D, log-transformed as a continuous variable, was independently associated with decreased risk of hospitalization.
2. Relative risk reflects 5 years increase in age.
3. Relative risk reflects 5% increase in proportion of residents in patient ZIP code living below federal poverty line.

**Appendix Table 6. Sensitivity Analysis – Including Serum Albumin with Independent Predictors of 60-day Mortality for 4,599** **Patients with Positive SARS-CoV-2 Tests (2/20/2020 – 11/8/2020)**

|  | Unadjusted |  | Age and Sex |  | Fully Adjusted |  |
| --- | --- | --- | --- | --- | --- | --- |
|  | RR/95% CI | p>\|z\| | ARR/95% CI | p>\|z\| | ARR/95% CI | p>\|z\| |
| 25-Hydroxy Vitamin D, log-transformed ^a^ | 1.09 (0.85,1.40) | 0.495 | 0.61 (0.47,0.79) | <0.001 | 0.69 (0.53,0.90) | 0.005 |
| Age at Index Date *^b^*, years |  |  | 1.47 (1.41,1.52) | <0.001 | 1.41 (1.34,1.49) | <0.001 |
| Male |  |  | 1.53 (0.88,2.65) | 0.133 | 1.31 (0.75,2.27) | 0.342 |
| **Low Serum Albumin** |  |  |  |  | **1.56 (1.25,1.95)** | **<0.001** |
| Race: Non-Caucasian or Unknown |  |  |  |  | 1.22 (0.98,1.52) | 0.073 |
| Ethnicity: Hispanic or Latinx |  |  |  |  | 0.97 (0.65,1.43) | 0.863 |
| Proportion of Residents < Federal Poverty Line *^c^* |  |  |  |  | 1.00 (0.94,1.05) | 0.867 |
| Body Mass Index, kg/m^2^ |  |  |  |  | 1.00 (0.98,1.02) | 0.957 |
| Diabetes (Any) |  |  |  |  | 1.40 (1.13,1.73) | 0.002 |
| Cardiovascular Disease (incl. Hypertension) |  |  |  |  | 1.25 (1.00,1.58) | 0.055 |
| Obstructive Sleep Apnea |  |  |  |  | 1.04 (0.83,1.30) | 0.741 |
| Chronic Obstructive Pulmonary Disease |  |  |  |  | 1.19 (0.96,1.47) | 0.104 |
| Cancer |  |  |  |  | 1.06 (0.86,1.31) | 0.569 |
| Chronic Kidney Disease |  |  |  |  | 1.54 (1.25,1.90) | <0.001 |
| Liver Disease |  |  |  |  | 1.03 (0.73,1.46) | 0.855 |
| Human Immunodeficiency Virus |  |  |  |  | 0.66 (0.16,2.68) | 0.565 |
| Current or Former Smoker |  |  |  |  | 1.06 (0.86,1.31) | 0.586 |
| Non-Alcohol Drug Dependence |  |  |  |  | 0.98 (0.55,1.73) | 0.933 |
| Alcohol Dependence |  |  |  |  | 1.27 (0.85,1.90) | 0.240 |

1. 25-Hydroxy Vitamin D, log-transformed as a continuous variable, was independently associated with decreased risk of 60-Day mortality.
2. Relative risk reflects 5 years increase in age.
3. Relative risk reflects 5% increase in proportion of residents in patient ZIP code living below federal poverty line.
